# Optimization of Modulation Methods for Solenoid Valves to Realize an Odor Generation System

**DOI:** 10.3390/s19184009

**Published:** 2019-09-17

**Authors:** Manuel Aleixandre, Kaoru Nakazawa, Takamichi Nakamoto

**Affiliations:** Institute of Innovative Research, Tokyo Institute of Technology, Yokohama 226-8503, Japan; manuel.aleixandre@gmail.com (M.A.); sc.cs.bundesu5280@gmail.com (K.N.)

**Keywords:** room temperature ionic liquid, gas sensor, odor generation system, e-nose, artificial olfactory system, calibration, Delta-Sigma modulator

## Abstract

An artificial olfactory system coupled with an odor generation system is herein reported. The artificial olfactory system is composed of four chemical sensors consisting of quartz crystal microbalances (QCMs) coated with room temperature ionic liquids (RTILs). The sensors are interrogated by four vector network analyzers, which are used to measure the series resonant frequency and motional resistance. The odor generation system can generate eight different odors and mix them in any composition. Solenoid valves are used to switch the path and control the concentration of the different odors before blending. Two algorithms to control the solenoid valves, delta-sigma modulator, and simple pulse width modulation (PWM) are studied, optimized, and compared. Finally, the uncertainty of the odor generating system is calculated.

## 1. Introduction

Human senses that detect chemical compounds, in particular scent and taste, have been traditionally left out of digitalization. However, efforts to include scent in electronic devices keep pushing knowledge, technology, and science, and also expanding the science of human olfaction, such as the effects on subjective perception or timing [1,2].

Systems designed to digitalize odors can be classified into two categories, recording and reproduction [3]. Recording is made by measurements that quantify compounds or identify odors [4]. The research in this area is mostly aimed at improving sensor sensitivity, selectivity, and stability, as well as improving systems by decreasing size, or improving algorithms [5,6].

The other category is an odor generating system. It consists of an odorous gas generator, a gas delivery system, and the electronics that control all parts. Depending on how the gas generation operates, it can be classified into different categories. The odor can be produced by vaporization (bubbling, thermal, air flow, etc.) or atomization (acoustic, spray, ink jet, etc.) of some odorant substance (essential oil, odorant volatile organic compounds, soaked porous materials, etc.). To generate complex odorant patterns, blending can occur before the odor generation takes place (mixing odorant liquids), or after the basic odors are generated (with mass flow controllers, or atomization of different sources) [3].

On the other hand, the systems designed to reproduce odors also present important challenges [2]. Some of them include odor persistency that consists of odors lingering in the fluidic system or in the environment. Materials such as Teflon can reduce odor persistency, and other strategies consist of reducing the volume of fluidic space. Another challenge is the repeatability of odor generation. A system that uses head space requires strict compound concentrations and precise timing of odor release, although variations of release times can be a problem when the irregular release of odors is required, or when the lifetime of an odor generation substance is shorter than the lifetime of the application. Other solutions use mass flow controllers or valves to control and mix flows. With all these issues, odor generation systems have multiple restrictions on their practical applications, and as a result devices are usually confined to laboratories and prototypes.

The blending system presented in this paper is based on earlier systems developed in a laboratory [7]. The odors are generated by air flow vaporization and mixing components before the odor presentation. This method has the advantages of being versatile as well as the possibility of digitally generating different scents on demand [8]. This system uses solenoid valves to generate concentrations. A concentration is regulated by the average duration of the solenoid valves on-time. Compared with mass flow controllers, solenoid valves have a cheaper cost (typically ~$70 compared to ~$1000), smaller size (typically ~20 cm^3^ compared to ~300 cm^3^), and lower power consumption (typically ~1 W compared to ~4 W), particularly when many are required to control multiple gas components channels. But the control of the solenoid valves requires more fine-tuned algorithms to avoid oscillations of the concentration at the outlet of the system. 

In [7], the system built could mix only two gas components, but the precision was not estimated. In [9], a simplification of the fluidic system facilitated the possibility of having 31 gas components, making that system more appropriate when many gas components were required. However, the flow rate had small variations with solenoid valve switching, and the error between gas components was estimated as 10%. Although the original method used delta-sigma modulation, a pulse width modulation (PWM) method was implemented in a previous approach due to its simplicity [10].

These devices were tested with gas sensors based on quartz crystal microbalances (QCM) interrogated by frequency counters. A frequency counter performs noise reduction owing to the integration during the frequency counter gate time (the pulses are counted over 1 s). If other parameters need to be extracted from the QCM beside the resonant frequency, other measurement strategies such as using vector network analyzers (VNA) are necessary, but if so then another method of noise reduction must be used.

This work studies two different algorithms, PWM and delta sigma modulation, to optimize them with the objective of decreasing the noise and uncertainty of the system below 10%, which was the previous estimate of the error between channels. Finally, a characterization of the system is performed to obtain a quantitative estimation of the gas concentration uncertainty that facilitates future comparison with other devices.

## 2. Materials and Methods

**Figure 1:**
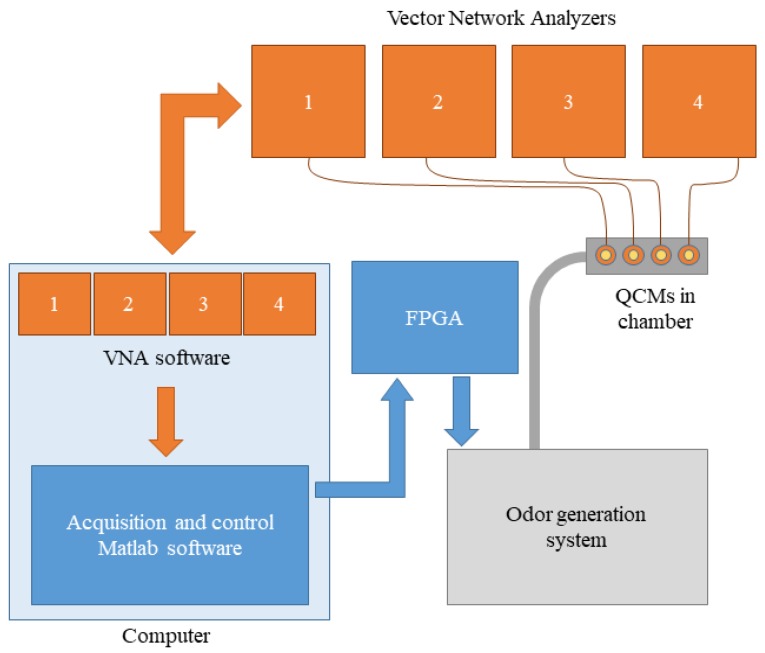
Scheme of the artificial olfactory system.

A brief explanation of the artificial olfactory system will be introduced first, including the quartz crystal microbalance (QCM) sensors and the reason needed to incorporate the VNAs. Next, a more detailed description of the odor generation system will be presented. Figure 1 shows a scheme of the whole system, including the artificial olfactory system and its integration into the odor generation system. The blue boxes represent the software and electronics used to control and record the measurements. The orange boxes represent the proprietary VNA software and electronics. The grey box represents the odor generation system block.

The odor generation system works independently of the odor sensor system. In this work, the sensor system was only used to characterize the odor generation system by measuring QCM sensor frequency shifts.

### 2.1. Artificial Olfactory System

**Figure 2:**
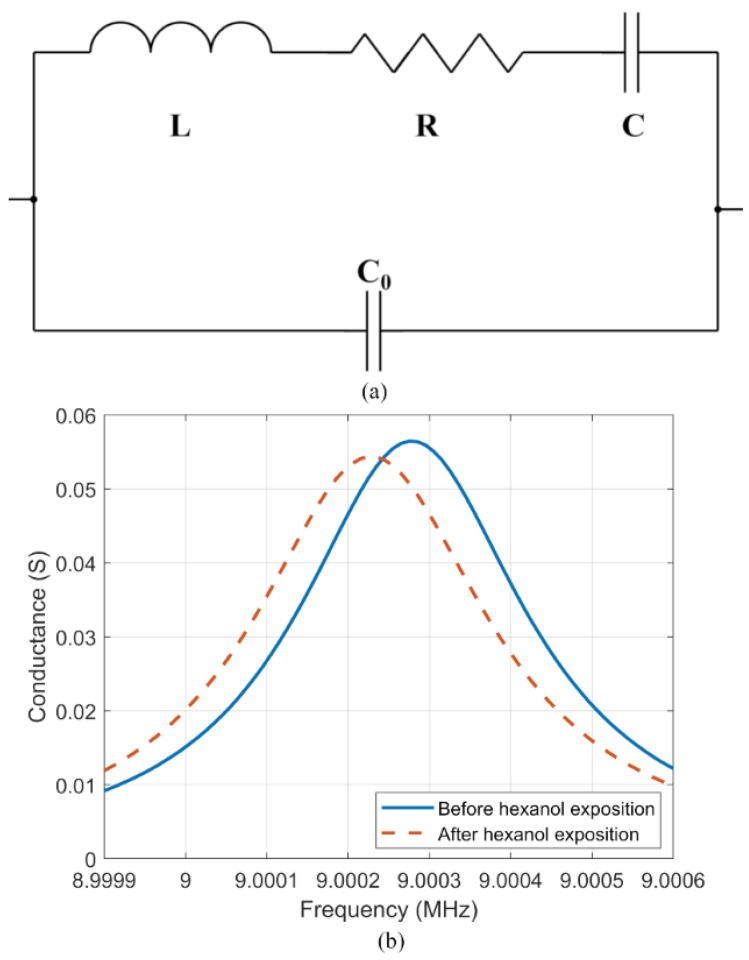
Behavior of the QCM. (**a**) Mason equivalent circuit. (**b**) Measured frequency characteristic for the QCM sensor coated with [BMIM][Cl] before (solid line) and after (dashed line) exposure to hexanol.

The sensor array in this artificial olfactory system consists of four QCMs placed inside a small chamber. QCM sensors allow quick and easy testing of different sensing materials just by depositing the sensing materials on the surface. The sensing material absorbs the gas and the resonant frequency of the QCM changes with the absorbed mass. The measurements of the QCM resonance characteristics are made by measuring a conductance curve (conductance vs frequency) then numerically optimizing parameters in a theoretical equivalent electric circuit, as shown in Figure 2a [11]. This calculation was made by MATLAB software.

This equivalent circuit is described by Equation (1), and it is a common way to characterize the QCMs in which *R* in the electrical circuit represents the loss in the physical device, *L* represents the mass loading of the QCM, and the series resonant frequency is given by Equation (2) [11]. Figure 2b shows two different measured frequency characteristics of one QCM before (blue solid line) and after (red dashed line) gas exposure.

(1) $$G=\frac{R}{R^{2}+{\left(2\pi fL-\frac{1}{2\pi fC}\right)}^{2}}$$

(2) $$f=\frac{1}{2\pi \sqrt{LC}}$$

The conductance curves were measured independently by four vector network analyzers (VNAs) (DG8SAQ VNWA v3), and each spectrum was measured every 2 s. VNAs work by running a proprietary software (DG8SAQ Version 36.7.6) in the computer that does the control and data recording. This software can communicate with other external software in diverse ways; in this work, they work by continuously measuring and dumping the data to text files. This method was more stable and generated faster measurements than interrogating or controlling the VNA software from external software.

Commercial QCMs (SEIKO EG & G, AT-cut) with a resonant frequency of 9 MHz were used to build the sensor array. According to the Sauerbrey equation (Equation (3))—where *f*_0_ is the resonant frequency, Δ*f* is the frequency shift, Δ*m* is the mass change, *A* is the electrode area, *ρ_q_* is the density of quartz, and *μ_q_* is the shear modulus of quartz—the frequency shift depends on the square of the resonant frequency, and because the frequency is relatively easy to measure, high-frequency QCMs are preferred.

(3) $$\Delta f=\frac{2f_{0}^{2}}{A\;\sqrt{\rho_{q}\mu_{q}}}\Delta m$$

The QCMs were coated with different room temperature ionic liquids (RTILs) by dip coating [12]. Before coating each QCM, its resonant frequency was recorded. Then the QCMs were submerged in a solution of the RTIL and a solvent (chloroform or acetone). The pullout speed was controlled by the dip coater and the final resonant frequency shift caused by the sensing layer (Δ*F_s_*) was recorded. The characteristics of the different sensors are described in Table 1.

The RTILs used in this work were 1-Methyl-3-n-octylimidazolium Bis(trifluoromethanesulfonyl)imide (abbreviated here as [MOIM][TFSI]), 1-Methyl-3-n-octylimidazolium Hexafluorophosphate ([MOIM][PF_6_]), 1-Butyl-3-methylimidazolium Chloride ([BMIM][Cl]), and 1-Butyl-3-methylimidazolium Bromide ([BMIM][Br]), as described in Table 1.

In addition to the mass loading and its influence on resonant frequency shift, the responses of QCMs coated with RTILs can also be characterized by the changes in RTIL viscosity. Unlike the more common approach of interrogating QCMs just by the resonant frequency with a frequency counter, the VNAs make it possible to simultaneously measure both changes on the series resonant frequency and resistance. This allows the measurement of viscosity effects, and although a QCM resonator does not work well under heavy viscous damping, VNA works even in that situation [12].

### 2.2. Odor Generation System

In this section, the hardware of the odor generation system is described, the algorithms used to control the valves are explained, the algorithms are analyzed and compared, and finally a short experimental validation is performed.

#### 2.2.1. Odor Generation System: Hardware

**Figure 3:**
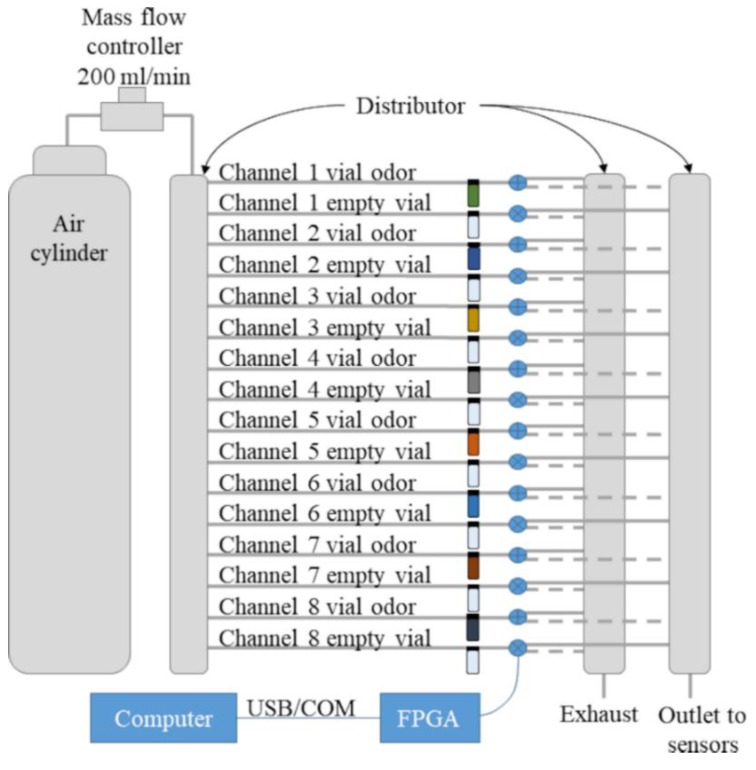
Scheme of the odor generation system.

**Figure 4:**
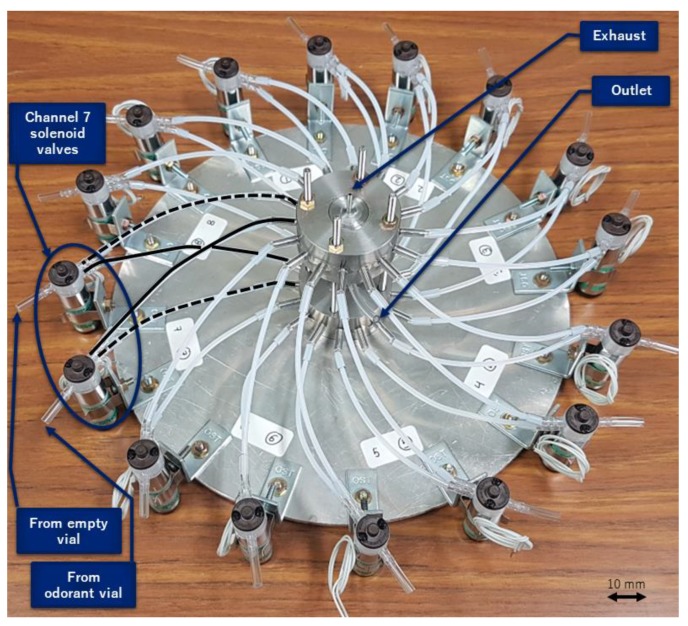
Detail of one assembly part of the odor generation system.

The odor generation system schematic can be seen in Figure 3. A cylinder of dry compressed air was the source of carrier gas and acted as zero gas. A mass flow controller (HoribaStec, SEC-400, Mark 3) regulated the flow rate at 200 mL/min. Then, the air flow reached a distributor that split the flow uniformly into 16 channels. The channels went to a rack of 16 vials of 10 mL, with half of the vials empty and the others containing 0.5 mL of an odorant liquid. Each vial was then connected to a three-way solenoid valve. The valves sent the flow that passed through the vial to either an outlet collector or an exhaust collector (we call these valve positions closed and open, respectively), and these paths are represented by dashed or solid lines, respectively, in Figure 3. The collector chambers were placed in a circular arrangement to make every channel length equal (Figure 4).

Each vial containing an odorant liquid was paired with an empty vial so that they alternated; when the flow of the channel with the vial containing the odorant liquid was directed to the exhaust, the flow of the paired channel with the empty vial was directed to the outlet, and vice versa. The flow was always passing through all paths in the system, so there were no flow rate or pressure changes. This arrangement can be seen in Figure 3. The distribution of channels and vials was used to maintain an equal path for each channel so that the flow was always constant and equal in each channel. Further, the gas concentration of each odor in its path remained constant, due to the constant flow passing through the vial. In this arrangement, each channel sent either the flow from the odorant vial or the flow from the empty vial to the outlet. Fast alternating of valves in a single channel can be used to control the average of the odorant concentration going through the outlet. In this scenario, all flows from all channels are added together at the outlet.

The valves (Takasago, EXAK-3) were controlled by a field-programmable gate array (FPGA, Altera Cyclone 5 SE 5CSEMA5F31C6N) that communicated with a computer through a USB COM port (chip: FT234XD-FTDI) interfaced with a computer. The FPGA accepted a command with a number from 0 to 100 for each channel that represented the percentage of odor flow going to the outlet, with 100 meaning that all the flow from the vial with the odorant was directed to the sensor chamber, and that the flow passing through the empty vial was directed to the exhaust. The FPGA read the command every second, setting new values for all channels at once. A transistor array was used to drive the solenoid valves.

The odor generation system tubing volume after the valves acted as a low pass filter (LPF) that mixed the gases. If the solenoid valve switching frequency was slow, the cutoff frequency of this filter was too low and noise would be seen by the sensors. To combat this, an optional buffer volume of 5 mL was implemented by a small vial at the outlet. This bigger volume acted as a LPF with lower cutoff frequency that smoothed the concentration changes generated by the alternating solenoid valves.

#### 2.2.2. Odor Generation System: Algorithms

In a preliminary test with a PWM algorithm, some noise was detected. The noise was caused by the abrupt change of the QCM conductance peak during the VNA frequency scan due to the on–off solenoid switching. Using a frequency counter, as in [7,9], the noise reduction can be automatically performed due to integration during the frequency counter gate time (in the previous works the peaks were counted over 1 s). However, VNAs are more sensitive to switching noise because they lack the proper integration mechanism, even when output space acts as a LPF.

To fix this problem, two different strategies were tested for the realization of the digital to analog converters (DAC) that transformed digital control signals into analog gas concentrations. The two strategies were different algorithms of the valve open/close commands—a simple pulse width modulation (PWM), and a delta-sigma modulator of first order [13]. The PWM has a fixed period with a variable duty width. This generates noise peaks at certain frequencies that are generally hard to eliminate using analog filters. On the other hand, the delta-sigma DAC has a variable duty cycle and a variable frequency, pushing the noise toward higher frequencies and also spreading the noise power more evenly. These effects make it easier to remove the noise via analog filtering [13]. 

The main constraint of the system was the switching speed of the valves, which was 15–20 ms when opening and 5–7 ms when closing. Valve control switching times that come close to these values will produce distortions in the desired final concentration percentages. Since the aim is to improve the accuracy of the system compared to former versions [10], the minimum pulse width used in the tests was set to the difference between the valve opening time and closing time (i.e., 0.01 s instead of 0.002 s).

The minimum pulse will be related to the frequency of the PWM and the delta-sigma modulator. For the PWM, because we had a resolution of 1%, the minimum pulse corresponded to a period of 0.01 s. For the delta-sigma modulator, the minimum pulse corresponded to the period of the algorithm. In the rest of this paper, we report the minimum pulse when comparing both algorithms.

#### 2.2.3. Odor Generation System: Analysis

**Figure 5:**
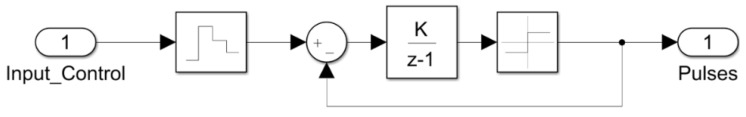
Simulink model of the delta sigma modulator.

To perform an exhaustive test on different configurations, a model of the odor generation system was made in Simulink. The model consisted of a single sensor coupled with the PWM or delta-sigma DAC, a model of the valves, and a model of a buffer volume that acted as an analog low pass filter (LPF). Figure 5 shows a Simulink model of the delta-sigma modulator in which the blocks from left to right are: input control, sampling and holding (used to change the sample rate), a subtraction of the output from the signal, an accumulator, a threshold quantizer that outputs zero or one, and the output of the subsystem. The state of the solenoid valve could change according to minimum pulse or in multiples of that time.

**Figure 6:**
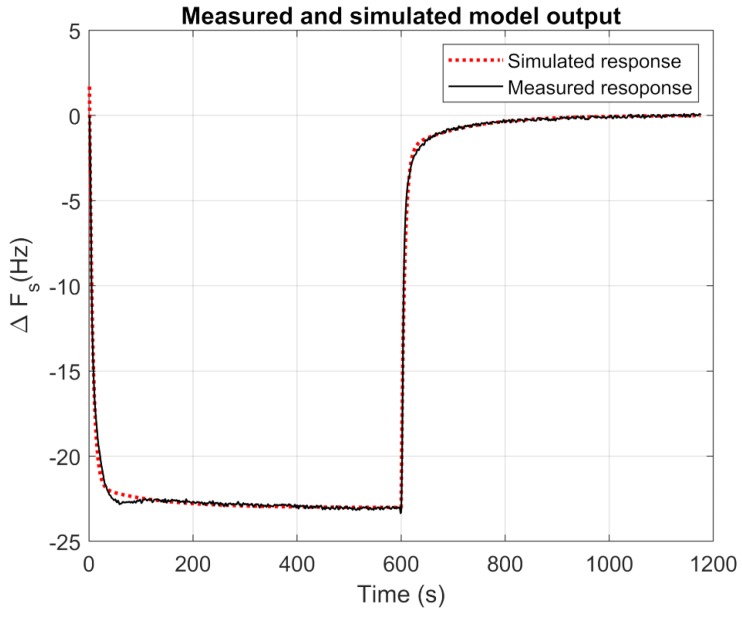
Response of a sensor at one step of hexanol (black) and its model, fitted as Equation (4) (dashed line) for the response of the [BMIM][Br] sensor to a hexanol step.

One sensor and one channel of the odor system tubing were modeled together as a second-order transfer function with two poles and one zero (Equation (4)). A model of second order is common in QCM as one time constant represents the speed at which the absorptions occur at the surface, and the other time constant represents the diffusion into the sensing layer. A response to a step from 0% to 100% of hexanol concentration, resampled to 1 Hz, was used to model the odor generation system and sensor, as shown in Figure 6. An adjustment made with the System Identification Toolbox of MATLAB yielded the following values: *a* = −0.03342, *b* = −0.0003197, *c* = 0.1673, and *d* = 0.001389.

(4) $$G=\frac{as+b}{s^{2}+cs+d}$$

The solenoid valves were manually modeled as a one-pole system (Equation (5)) with *e* = 462 when closing and *e* = 177 when opening, values that yielded 90% of the response at 17.5 and 6 ms, respectively. Also, because of these different switching times for the opening and closing valves, a compensation was implemented in the Simulink model by adding a delay to the valve closing, making the time intervals equal. That delay was also added to the FPGA control circuits.

(5) $$G=\frac{e}{s+e}$$

An optional buffer volume of 5 mL (implemented by a small vial) at the outlet, which was acting as an LPF, was modeled as a first-order transfer function (also Equation (5)) where *e* = 3 was given by the volume (V) and flow rate (F) as *a* = V/F [14].

The model was used to perform several tests, comparing the different strategies and optimizing the values of the control algorithms. Several frequencies for the PWM and delta-sigma modulation were tested, the maximum frequencies produced pulses widths of 0.01 s. of 0.01 s. The system was tested with target concentrations from 10% to 90%. The simulation time was 10 min and the last 60 s of simulation were used to calculate the noise. Two different signal-to-noise ratios (SNRs) were used to characterize the system. The first was the SNR_q_ corresponding to the quantization caused by the PWM or delta-sigma DAC, as given by Equation (6), where *S* is the mean of the simulated signal and *σ* is the standard deviation of the simulated signal. When the minimum pulse gets shorter, the sensors have less time to respond and to follow the ups and downs of the concentration. In this scenario, the sensor oscillation decreases its amplitude, and the SNR gets higher.

(6) $$SNR_{q}=10{\;\log}_{10}\left(\frac{S^{2}}{\sigma^{2}}\right)$$

The second SNR, SNR_dis_, corresponded to the distortion caused by the difference of the valve switching times. It was defined as the difference between the ideal response and the mean response obtained, and given by Equation (7), where *S_ideal_* is calculated from the specified concentration ratio percentage multiplied by the response at 100% (where the control system keeps the valve totally open), and Δ*S* is the difference between that ideal signal and the simulated signal. Also, the quantization noise will produce some oscillations in the signal, which will also contribute to the reconstruction noise. At some algorithm frequencies, this contribution to the noise will be greater than the contribution coming from the valves.

(7) $$SNR_{dis}=10{\;\log}_{10}\left(\frac{S_{ideal}^{2}}{\Delta S^{2}}\right)$$

This *SNR_dis_* is lower as we approach shorter minimum pulses, because the errors caused by the valve operation time difference become more important. The combined SNR, expressed in decibels, is given by Equation (8), in which *S_ideal_* is the ideal response, and the noise is calculated as the root sum squared of σ and Δ*S* errors [15].

(8) $$SNR=10\;\log_{10}\left(\frac{S_{ideal}^{2}}{\sigma^{2}+\Delta S^{2}}\right)$$

**Figure 7:**
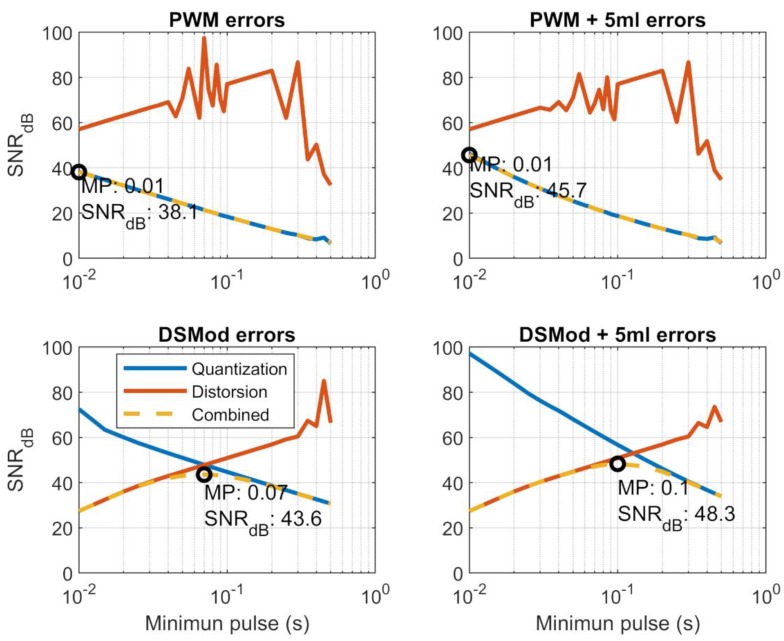
Errors of reconstruction in the different control strategies tested. The black circles represent the optimum minimum pulse (MP).

The results of the simulations, sampled at 1 ms, can be seen and compared in Figure 7. Plots titled PWM show the SNR of the PWM algorithm, plots titled DSMod show the sigma-delta modulator SNR, and titles including “+5 mL” indicated that the buffer volume at the outlet is included. The orange line represents the *SNR_dis_* that originated from the distortion, and we can see that it becomes worse as the minimum pulse decreases and the effects of the alternating valves start to have more weight in the signal. On the other hand, the SNR_q_ that originated from the quantization noise increases with the frequency (blue line), and at higher frequencies the sensor acted as an LPF that smoothed the signal, lessening the quantization noise. The oscillations of the *SNR_dis_* in the PWM were produced by the oscillation noise, which occurs when the signal oscillates between the valve in on and off states. Depending on the time at which the average measurement is taken (just after the valve opened or after the valve closed), this error can vary. In Figure 7, the combined SNR is drawn in yellow (sometimes overwriting the blue or orange lines), and the optimum value is represented by a black circle in the plot.

For the PWM and delta-sigma modulator, the addition of the buffer volume increased the SNR of the quantization errors. For the PWM control with buffer space, the optimal minimum pulse was 0.01 s, which corresponded to a PWM duty cycle of 1 Hz and had a SNR of 45.7 dB. For the delta-sigma DAC with buffer space, the minimum pulse was 0.1 s, which corresponded to a frequency of 10 Hz, and the SNR was 48.3 dB, which was better than the PWM. This was caused by a better balance between the reconstruction error and quantization error.

**Figure 8:**
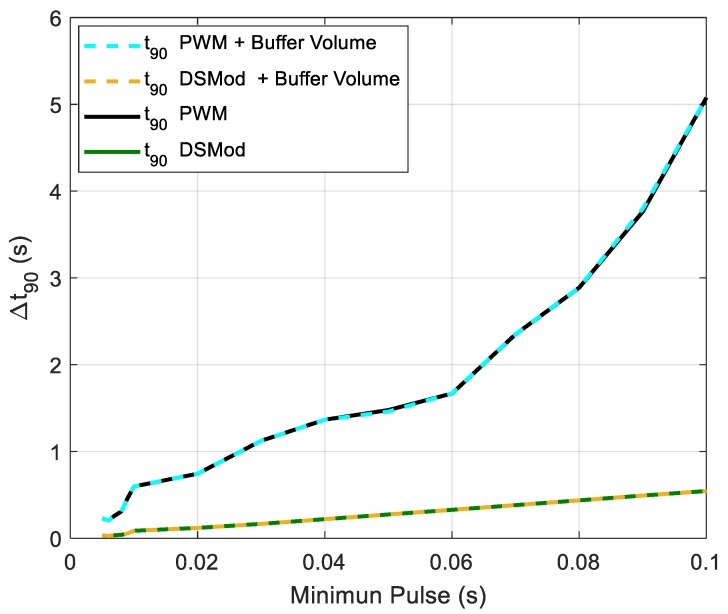
Differences of the *t*_90_ for the different control simulated strategies.

Another aspect to consider is the time that the system takes to reach the stationary state. We defined *t*_90_ as the time the system takes to reach the 90% of the final response, and the same model was used to calculate this time for all the configurations of the control modes. The target concentration percentage was swiped between 10% and 90%, then the *t*_90_ was averaged. The minimum pulse was swiped between 0.01 and 0.1 s. The averaged *t*_90_ for both PWM and delta-sigma were very similar (with and without buffer volume at the outlet), taking the values of 32 s for a minimum pulse of 0.01 s, and 42 s for a minimum pulse of 0.1 s. However, there was a difference, as the PWM control produced a higher difference between the maximum *t*_90_ and minimum *t*_90_ than the delta-sigma modulation. This can be seen in Figure 8, where the differences between maximum *t*_90_ and minimum *t*_90_ among the different concentration levels (10%–90%) versus the pulse width are drawn.

This lower difference of the delta-sigma modulator was caused by its more evenly distributed on times compared with the PWM. This means that the time to reach the steady state varied more with the frequency for the PWM control than for the delta-sigma modulation control.

#### 2.2.4. Odor Generation System: Validation

**Figure 9:**
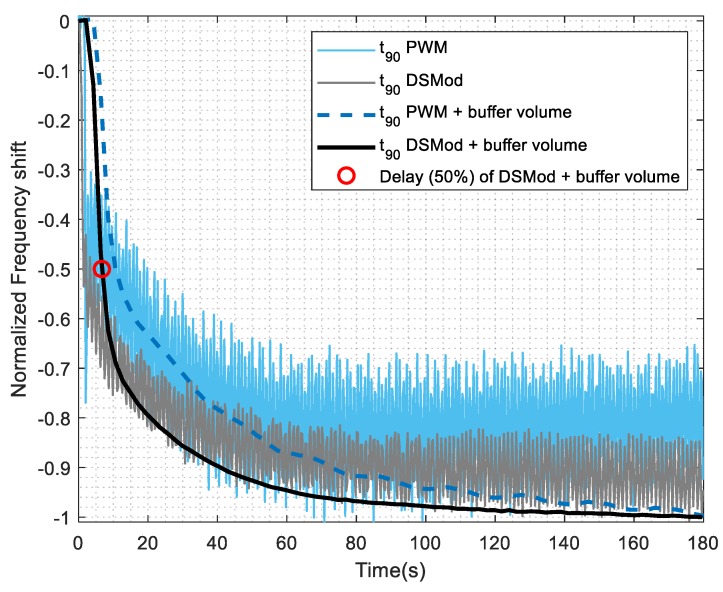
Effect of the different control strategies for the [BMIM][Br] sensor to hexanol at 50% controlled concentration percentage. Conditions of the measurements were made at a minimum pulse of 0.01 s for PWM and a minimum pulse of 0.1 s for the delta-sigma modulator, and a buffer volume of 5 mL was used.

The different control strategies were tested in the real system and the results can be seen in Figure 9. An effect is clearly seen and the delay calculated (as the time at which the signal falls 50%, shown in the plot as a red circle) as 6 s. The delta-sigma modulation DAC with a buffer volume of 5 mL at the outlet was selected to implement the real system because it had a high SNR and the *t*_90_ had less variability.

## 3. Results

With the system optimized, several tests were used to characterize it. In the first subsection, the odor generation system concentration levels and sensor sensitivity are calculated. Then, the uncertainty of the odor generation system is calculated.

### 3.1. Concentration and Sensitivity

**Figure 10:**
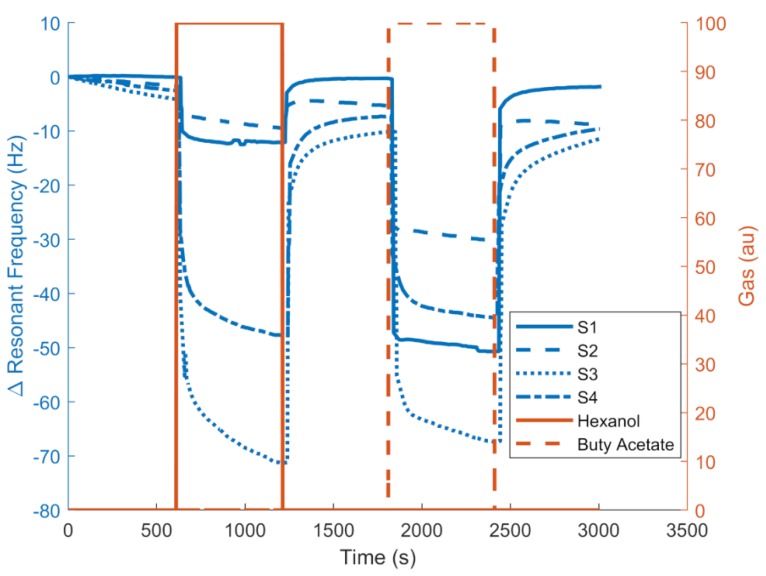
Example of frequency response of the [MOIM] [PF_6_] sensor for hexanol and butyl acetate.

**Figure 11:**
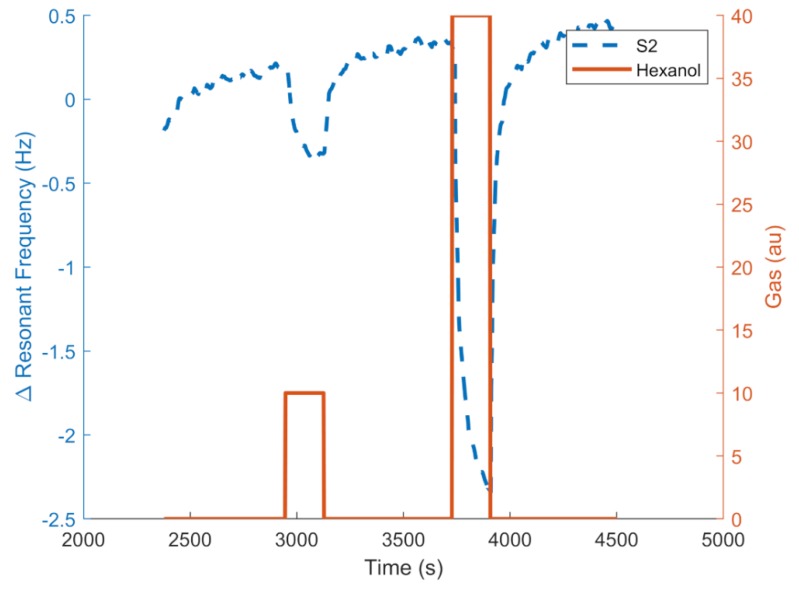
Example of frequency response of the [MOIM] [PF_6_] sensor for to two hexanol concentrations.

Figure 10 and Figure 11 show an example of the responses (resonant frequency shift) of the QCMs coated with different RTILs. Figure 10 shows the responses to hexanol and butyl acetate at maximum concentrations, showing that the sensors had different responses and a potential to distinguish between the two gases. The QCM frequency shift is represented in the left vertical axis, and the control target concentration is represented in the right axis. Figure 11 shows the responses in frequency of the S2 sensor to different hexanol concentrations generated by the odor generation system with the optimal conditions determined (0.1 s minimum pulse delta-sigma modulator with a 5-mL buffer volume).

The gas concentration in parts per million in volume (ppmv) of the gases were calculated by the mass change in the vials containing the odorant liquids, the duration of the experiments, and the flow rate passing through them. Two gases with different vapor pressures, hexanol with a concentration of 63 ppmv, and butyl acetate with a concentration of 760 ppmv were used. Using this we can calculate the sensitivities of the QCM sensors used in the system, which are shown in Table 2.

Several experiments were performed to characterize the uncertainty of the odor generation system and the noise of the artificial olfactory system measurements. For the latter, measurements of the line base for periods of time similar to one measurement cycle (around 15 min) were performed to estimate noise. For the odor generation system, the responses of one QCM were used to estimate the overall uncertainty of the system.

### 3.2. Precision of the Odor Generation System

To estimate the noise of the frequency and resistance measurements, the four sensors were measured for 900 s. Next, a polynomial fit to the measurements was made and the root mean square error (RMSE) for each sensor was calculated from the residuals. The averaged noise was 0.005 Hz for the frequency measurements, and 0.05 ohms for the resistance. This does not include noise from the temperature changes or fluctuations at the sensing layer. 

Because the software of the VNAs was running in Windows, the sampling time accuracy was also studied. The same experiments used to estimate noise were used to measure the time intervals between data points, with a sampling period of 2.126 ± 0.016 s. In any case, there is a need for resampling if any frequency analysis is to be done.

The frequency, resistance error, and period variability of the measurements were very low, and did not contribute much to the overall uncertainty of the system.

The odor generation system was also tested to check the linearity, noise, and difference between channels. While the system was able to blend different compounds, the same odorant was used in all vials, and only one channel was active at a time, so only single channels were calibrated. Since the channels worked independently, the final output was a superposition of all channels. These measurements consisted of exposures to control target concentrations from 0% to 100% in 10% steps made in random order (of the eight channels and the 10 control target concentrations). The exposure time was 3 min, and the recovery time after each exposure was 10 min. All measurements were taken with the sensor S1 from the Table 1.

This set of measurements was repeated twice under the same conditions, with the first one used to calibrate, and the second one to validate the calibration and estimate the uncertainty, a procedure that was used to avoid overfitting. The uncertainties were calculated following the indications in [15], and they give an idea of the precision of the overall system. To calculate these uncertainties, the first dataset was used to make a linear regression of the frequency versus the control target concentration. That regression was then used with the second dataset to predict a relative concentration. The predicted concentration and control target concentration of the second dataset were used to calculate an orthonormal regression using Equation (9), where *x* is the control concentration percentage, *y* is the predicted concentration percentage, and *a* and *b* are the calculated linear orthonormal regression coefficients.

(9) Y=a+bX

Next, the uncertainty was calculated using Equations (10) and (11), where n is the number of measurements, *U* is the uncertainty at the maximum control variable, and *X_max_* is the maximum control variable.

(10) $$U^{2}=\frac{RSS}{\left(n-2\right)}+{\left[a+\left(b-1\right)X_{max}\right]}^{2}$$

(11) $$RSS=\overset{n}{\underset{i=1}{\sum }}{\left(Y_{i}-a-bx_{i}\right)}^{2}$$

Table 3 shows the calculated uncertainties and root mean square error (RMSE) of every channel.

**Figure 12:**
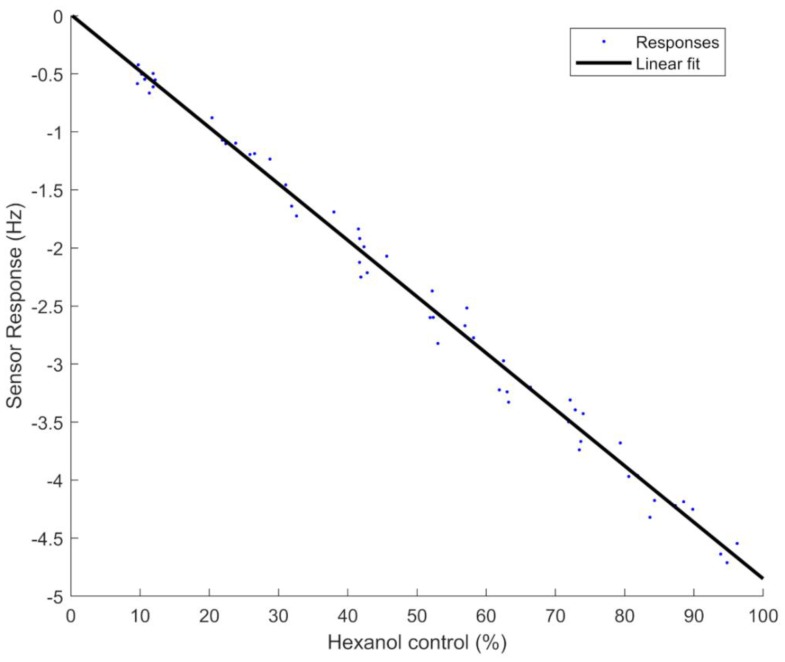
Linear adjustment of all compensated concentration percentages produced by all channels.

These scales then were used to do a linear calibration of each channel and compensate the differences among channels. Equation (12) was used to perform this compensation, where $X_{i}^{\prime }$ is the compensated control target concentration, $X_{i}$ is the control target concentration, $b_{i}$ is the slope of each channel, $b_{min}$ is the minimum slope among all channels, and $a_{i}$ is the intercept of the *I* channel, all of which were calculated with the first set of measurements. Figure 12 shows the second set of measurements compensated by these linear adjustments based on Equation (9). The uncertainty of the gas concentration with this compensation was 2.2%, and the RMSE 2.5%.

(12) $$X_{i}^{\prime }=X_{i}\cdot \frac{b_{min}}{b_{i}}-a_{i}$$

## 4. Discussion

An odor generation system based on continuous flow head space and solenoid valves coupled with an array of four QCM gas sensors has been presented. The current work uses the configuration of [7], although the number of gas components has been increased to eight. The odor generation system in [9] was made up of 32 solenoid valves with only one empty vial, which is more appropriate when many gas components are used since many empty vials are not required, but the flow rate through the vials had small variations, because of alternating solenoid valves, and the error between channels was estimated as 10%. In those previous systems, oscillation frequencies of QCM sensors were measured by frequency counters. In this work, on the other hand, the QCM frequencies were measured by VNAs that measure their conductance curves, allowing us to measure both the resonant frequency and resistance. However, this method is more sensitive to noise, so an optimization of the odor generation system was needed.

The optimization of the odor generation system determined that the optimal control strategy was a delta-sigma modulation with a frequency of 10 Hz and a small buffer volume at the outlet of 5 mL acting as an additional LPF. With this setup, the estimated uncertainty was below 3%, which is the typical uncertainty required for pollution measurements [16] and is well below the uncertainty in olfactometry devices (25%), although in this case the concentration range was much reduced [17]. In contrasts with previous systems [9], the uncertainty of the current odor generation system was below 2.5%.

These low RMSEs and uncertainties include the noise of the delta-sigma DAC and also the head space dependence on ambient temperature. The measurements took around 37 h, which spanned temperature variations between day and night. Wider temperature changes would increase the RMSE and uncertainties, while a tighter control of the temperature in the vials could decrease them.

With this controlling algorithm (delta-sigma modulation with minimum pulse of 0.1 s), the system completes a cycle in 1 s when the target control concentration is 10%. However, the system has been shown to reach a stationary state after around 40 s, which is not fast enough for applications such as real-time odor displays (human perception time is around 2.5 s [18]), but is sufficient for odor generation in sensor array testing (chemical sensors typically have longer response times [19]). Faster responses would be preferable.

Since the system works together with an artificial olfactory one, it offers the possibility of measuring numerous samples with very complex aroma profiles. Furthermore, the artificial olfactory system measures the resonant frequency and series resistance of the QCM simultaneously by vector networks analyzers. This allows the extraction of additional information from the QCM to improve future classification tasks.

Future works for improving odor generation systems could include higher-order delta-sigma modulators or dynamic adaptation of the minimum pulse of the algorithms to increase response speed and accuracy. The versatility and possibility of blending eight different channels with this system would allow the characterization of sensors with very complex odor patterns and the testing of advanced odor recognition algorithms.

## Figures and Tables

**Table 1:** QCM coating used in this paper.

Sensor	S1	S2	S3	S4
Coating Material	[MOIM][TFSI]	[MOIM][F_6_]	[BMIM][Cl]	[BMIM][Br]
Solvent	Acetone	Acetone	Chloroform	Chloroform
Concentration (gr/mL)	0.0203	0.0147	0.0100	0.0104
Dip coating speed (μm/s)	50	50	50	50
Sensing layer ΔFs (Hz)	2264	1277	3486	2460

**Table 2:** Sensitivities of the QCM plus RTIL sensors to hexanol and butyl acetate in Hz/ppmv.

	S1	S2	S3	S4
Hexanol	0.192	0.151	1.132	0.760
Butyl Acetate	0.067	0.040	0.100	0.059

**Table 3:** Estimated uncertainties (U) of the gas generation system.

	Ch1	Ch2	Ch3	Ch4	Ch5	Ch6	Ch7	Ch8
RMSE (%)	2	2.4	0.8	1.2	1.6	1.1	3.0	1.0
U (%)	2.1	2.3	1.3	1.7	1.9	1.6	2.6	1.5
